# Increased methylation upstream of the *MEG3* promotor is observed in acute myeloid leukemia patients with better overall survival

**DOI:** 10.1186/s13148-019-0643-z

**Published:** 2019-03-15

**Authors:** Zachariah Payne Sellers, Lukasz Bolkun, Janusz Kloczko, Marzena Liliana Wojtaszewska, Krzysztof Lewandowski, Marcin Moniuszko, Mariusz Z. Ratajczak, Gabriela Schneider

**Affiliations:** 1 Stem Cell Institute at James Graham Brown Cancer Center, University of Louisville, Louisville, KY USA; 2 Department of Hematology, Medical University of Bialystok, Bialystok, Poland; 3 Department of Hematology and Bone Marrow Transplantation, University of Medical Sciences, Poznań, Poland; 4 Department of Allergology, Medical University of Bialystok, Bialystok, Poland; 5 Department of Regenerative Medicine and Immune Regulation, Medical University of Bialystok, Bialystok, Poland; 6 Department of Regenerative Medicine, Medical University of Warsaw, Warsaw, Poland

**Keywords:** *DLK1*-*MEG3*, Imprinting, Leukemia, Cancer stem cells, miRNAs

## Abstract

**Background:**

The delta-like non-canonical Notch ligand 1 (*DLK1*)-maternally expressed 3(*MEG3*) locus (*DLK1*-*MEG3* locus) plays a critical role in the maintenance and differentiation of hematopoietic stem cells. Accumulating evidence implicates the imprinted genes from this locus, *DLK1* and *MEG3*, in the development and progression of acute myeloid leukemia (AML). However, the contribution of this locus to the treatment response of patients and their survival is unknown.

**Methods:**

DNA methylation of select CG dinucleotide-containing amplicons (CpG sites) within the *DLK1*-*MEG3* locus and within differentially methylated regions of other imprinted loci was assessed in the mononuclear cells of 45 AML patients by combined bisulfite restriction analysis. Methylation results were compared with patient response to first-round induction therapy and overall survival. Multivariable analysis was employed to identify independent prognostic factors for patient overall survival in AML.

**Results:**

Increased methylation at CpG sites within the *MEG3* promotor region was observed in AML patients having longer overall survival. In addition, patients with shorter overall survival had increased expression of *DLK1* and *MEG3*, and methylation at the *MEG3*-DMR CpG site inversely correlated with *MEG3* expression. Multivariable analysis revealed that methylation at CG9, a non-imprinted CpG site within the *MEG3* promotor region which contains a CCCTC-binding factor (CTCF)-binding DNA sequence, is an independent prognostic factor for the overall survival of AML patients.

**Conclusions:**

The results of our pilot study underscore the importance of the *DLK1*-*MEG3* locus in AML development and progression. We identify CG9 methylation as an independent prognostic factor for AML patient survival, which suggests that distinct miRNA signatures from the *DLK1*-*MEG3* locus could reflect varying degrees of cell stemness and present novel opportunities for personalized therapies in the future. These data provide a foundation for future studies into the role of higher-order chromatin structure at *DLK1*-*MEG3* in AML.

**Electronic supplementary material:**

The online version of this article (10.1186/s13148-019-0643-z) contains supplementary material, which is available to authorized users.

## Introduction

Genomic imprinting is an epigenetic process governed by complementary chromatin structures inherited from the mother and father. With the help of long non-coding RNAs (lncRNAs), differential covalent modifications of maternally and paternally inherited DNA and histone proteins control certain gene dosages in a parent-of-origin-specific manner [1]. The human genome contains more than 100 such imprinted genes which cluster around CG-rich regions of DNA. These regions, known as differentially methylated regions (DMRs), exhibit unique patterns of methylation at CG cytosine residues based on the parental origin of the chromosome. The temporo-spatial dosage of imprinted genes governed by genomic imprinting is integral to proper growth and development, and its dysregulation is found in several developmental abnormalities [2] and malignancies such as leukemia [3].

Embryonic and postnatal growth is under the control of a select group of coregulated imprinted genes which comprise an imprinted gene network (IGN) [4, 5]. Genes that belong to the IGN are highly expressed during embryonic and early postnatal development, but they are downregulated during maturation as the somatic growth rate decelerates [4]. Interestingly, targeted deletion of the lncRNA *H19*, a member of the IGN, results in the overexpression of other IGN genes [6–8] and postnatal overgrowth [8], both of which normalize upon transgenic *H19* re-expression [6, 7]. Similarly, a pivotal work in oocyte fusion revealed that genomic imprinting at the paternally imprinted *Igf2*-*H19*, which results in *H19* overexpression in bimaternal embryos, prevents the growth and development of bimaternal mice [9], highlighting *Igf2*-*H19* manipulation as a master switch which allows for parthenogenesis. However, it was soon realized that a second paternally imprinted locus housing IGN genes, *Dlk1*-*Meg3*, was also responsible for the restricted growth and development of bimaternal embryos [10], and a high efficiency of bimaternal mouse generation was later achieved via genetic manipulation mimicking somatic imprinting of both loci [11].

The *IGF2*-*H19* and *DLK1*-*MEG3* loci each contain oppositely imprinted tandem sets of IGN genes. The *IGF2*-*H19* locus encodes the growth-promoting protein-coding insulin-like growth factor 2 (*IGF2*) and lncRNA *H19*, and similarly, the *DLK1*-*MEG3* locus encodes the growth-related protein-coding delta-like non-canonical Notch ligand 1 (*DLK1*) and tumor suppressor lncRNA *MEG3*. Both loci also house growth-related microRNAs (miRNAs), and genomic imprinting may be similarly orchestrated for these loci by a single intergenic DMR found within each locus [12]. Interestingly, high expression of several IGN genes, including those located within *Igf2*-*H19* and *Dlk1*-*Meg3*, is restricted to hematopoietic stem cells (HSCs) in the adult [4, 13]. Moreover, the *Igf2*-*H19* and *Dlk1*-*Meg3* loci are uniquely important to HSC fate, as they harbor IGN genes which are required for fetal hematopoiesis [14] and miRNAs which govern HSC quiescence [15] and stemness [16].

Accumulating evidence suggests that a direct lineage can be drawn between primordial germ cells (PGCs) and adult HSCs [17], suggesting that HSCs retain PGC-like epigenetic programming for quiescence and stemness maintenance. In this regard, recent studies found that the *IGF2*-*H19* and *DLK1*-*MEG3* loci are involved in the stemness maintenance, proliferation, and tumorigenic potential of embryonal carcinoma (EC) cells [18–20], the cancer stem cells responsible for teratoma formation [21], implicating these loci in the malignant transformation of PGCs to EC cells during PGC maturation. Similarly, aberrant imprinting and expression of IGN genes including those from the *IGF2*-*H19* and *DLK1*-*MEG3* loci occurs in leukemias, leading to the hypothesis that very small embryonic-like stem cells (VSELs), as the pluripotent stem cells atop the adult stem cell hierarchy, may adopt a malignant fate along their differentiation route to HSCs via improper epigenetic transitions [3]. Thus, VSELs potentially represent both the adult stem cell linking PGCs to HSCs [17] as well as a source of preleukemic HSCs, where epigenetic control over *IGF2*-*H19* and *DLK1*-*MEG3* is critical to the maintenance of a healthy HSC compartment.

Hypomethylation of the *IGF2*-*H19* DMR with concomitant-elevated *H19* expression is characteristic to VSELs and hypothesized to control their quiescence [22]. In support of this, VSEL expansion strategies are accompanied by de novo methylation of the *IGF2*-*H19* DMR and the emergence of differentiated cells [22, 23]. Interestingly, *H19* overexpression is important for leukemic cell proliferation, and DMR methylation-independent *H19* overexpression predicts poor survival in AML [24]. Similarly, *DLK1* overexpression prevents leukemic cell differentiation [25], and *DLK1* is overexpressed in AML via aberrant methylation of an upstream insulator region [26]. On the other hand, *MEG3* inhibits leukemogenesis [27] and leukemic cell proliferation [28], but its expression is decreased in AML samples [27, 29] and inversely correlates with promotor methylation [29]. Furthermore, the *DLK1*-*MEG3* miRNA mega-cluster is known to regulate cell stemness [16, 30, 31] and self-renewal [16, 32], and the expression of these miRNAs correlates with methylation at CpG sites throughout this locus in acute promyelocytic leukemia (APL) [33]. Taken together, in contrast to the methylation-independent expression of *H19* [24], it appears likely that aberrant methylation within *DLK1-*MEG3 underlies gene dysregulation from this locus and represents a source of potential prognostic factors for AML survival.

**Fig. 1:**
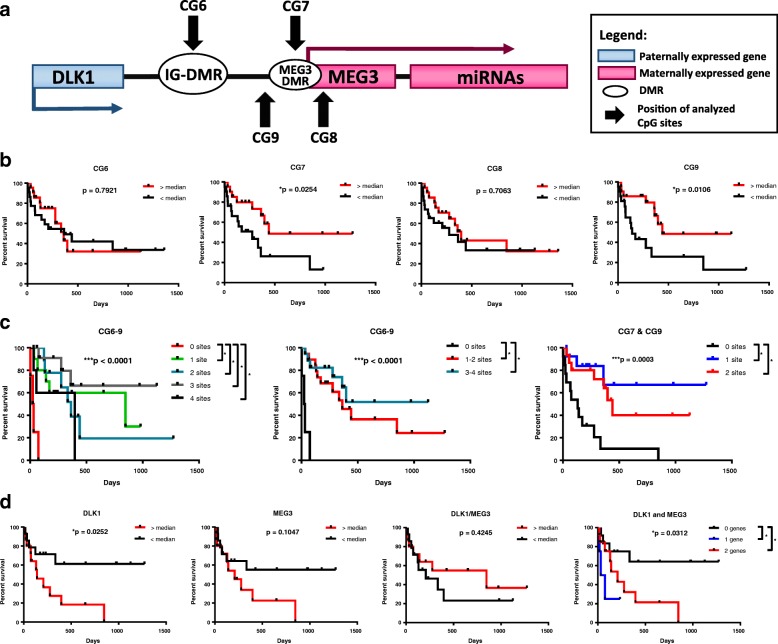
Survival of AML patients based on *DLK1*-*MEG3* CpG site methylation and imprinted gene expression in AML MNCs. **a** Schematic representation of the human imprinted *DLK1-MEG3* locus. **b** Analysis of AML patients’ survival depending on median methylation values for particular CpG site amplicons using the Mantel-Cox test. *p < 0.05. Sample counts for “< median”: CG6 *n* = 21, CG7 *n* = 20, CG8 *n* = 22, and CG9 *n* = 20; and for “> median”: CG6 *n* = 22, CG7 *n* = 21, CG8 *n* = 23, and CG9 *n* = 21. **c** Analysis of AML patients’ survival depending on pooled CG6–CG9 or CG7 and CG9 amplicon median methylation values using the Mantel-Cox test. Lines represent the number of CpG site amplicons out of 4 (CG6–9) or 2 (CG7 and CG9) analyzed which exhibited increased methylation relative to their respective median values. ***p < 0.001 and Bonferroni post hoc analysis *p < 0.05. Sample counts for CG6–9 (0–4 sites): 0 site *n* = 3, 1 site *n* = 10, 2 sites *n* = 9, 3 sites *n* = 13, and 4 sites *n* = 5; for CG6–9 (0, 1–2, 3–4 sites): 0 site *n* = 3, 1–2 sites *n* = 19, 3–4 sites *n* = 18; and for CG7 and 9: 0 site *n* = 12, 1 site *n* = 14, and 2 sites *n* = 15. **d** Mantel-Cox analysis of AML patients’ survival depending on median *DLK1*, *MEG3*, and *DLK1*/*MEG3* and pooled *DLK1* and *MEG3* expression ratio values obtained by RT-qPCR. Red lines represent patients with increased expression of *DLK1* and *MEG3* and an increased *DLK1*/*MEG3* ratio. For pooled expression results, the Bonferroni post hoc analysis was used and lines represent the number of genes which exhibited increased expression relative to their respective median values. *p < 0.05. Sample counts for “> median” expression: DLK1, MEG3, and DLK1/MEG3 *n* = 14; and for “<median” expression: DLK1, MEG3, and DLK1/MEG3 *n* = 15

To this end, we assessed methylation at select CpG sites within *DLK1*-*MEG3* (Fig. 1a) and imprinted gene expression from this locus in the peripheral blood mononuclear cells of AML patients. We then compared our results with patient outcomes to probe for independent markers of prognostic value. Based on correlations between CpG site methylation and miRNA expression from this locus in APL, in addition to the roles for this locus in HSC stemness maintenance and leukemic cell proliferation, we hypothesized that patients’ response to chemotherapy and overall survival would depend on CpG site methylation and imprinted gene expression from the *DLK1*-*MEG3* locus.

## Methods

### Study design and description of patient samples

This investigational pilot study was designed to evaluate the impact of imprinting within *DLK1-MEG3* locus on survival and response to treatment of patients with acute non-promyelocytic leukemia (AML). Forty-five patients with newly diagnosed AML were included in the study. Both patients with acute promyelocytic leukemia, due to the specific biology and a different outcome, as well as AML patients who received corticosteroids at the beginning of the treatment course, were not included in the study. Diagnoses were established following the WHO classification system [34]. Blood counts and flow cytometry were performed to confirm the presence of blastic cells, whereas cytogenetic and molecular studies, including the FISH study (*AML1*/*ETO*, *CBFß*/*MYH11*, and *MLLT3*-*MLL* and frequently mutated genes *FLT3*-*ITD*, *NPM1*, and *CEBPA*), were performed to determine the risk group, according to the WHO recommendation. On the basis of the above, patients were classified as follows (Table 1): 7 (15.6%) patients had good risk (1 patient with t(8;21), 4 with inv. [16]/t(16;16), and 1 with mutated CCAAT/enhancer-binding protein alpha (*CEBPA*_*mut*_)), 10 (22.2%) subjects had 1st intermediate risk (diploid karyotype features with 2 having both mutated nucleophosmin (*NPM1*_*mut*_) and internal tandem duplication of FMS-like tyrosine kinase 3 (*FLT3-ITD*) and 7 with *FLT3-ITD* without *NMP1*_*mut*_), 18 (17.8%) patients had 2nd intermediate risk (3 patients with t(9;11) and 7 with different abnormalities not assigned to either good or bad risk group), and 20 subjects (44.4%) were classified as unfavorable risk group with del(5q), del(7q), or complex (≥ 3) abnormalities. Patient information is listed in Table 1.

**Table 1:** AML patient information

Parameter		Characteristic	Value
General Information		Number of patients	45
		Mean (range) age in years	48.7 (19–65)
		Mean (± SD) white blood cell count (G/l)	45.01 ± 65.95
		Mean (range) of blastic cells in the peripheral blood (%)	56 (0–97)
		Mean (range) of blastic cells in the bone marrow (%)	65 (20–97)
		Mean (± SD) lactate dehydrogenase (U/L)	504.4 ± 344.4
AML subtypes based on WHO classification (*n*,(%))	AML with recurrent genetic abnormalities	t(8;21)(q22;q22);(*AML1*/*ETO*)	1 (2.2%)
		inv [16](p13;q22) or t(16;16)(p13;q22);(*CBFβ*/*MYH11*)	4 (8.9%)
		t(9;11); *MLLT3*-*MLL*	2 (4.4%)
	AML with multilineage dysplasia without antecedent MDS		3 (6.7%)
	AML (therapy-related)		0 (0%)
	AML (not otherwise categorized; *n* = 35)	AML (minimally differentiated)	4 (8.9%)
		AML (without maturation)	8 (17.8%)
		AML (with maturation)	13 (28.9%)
		Acute myelomonocytic leukemia (AMMoL)	8 (17.8%)
		AMMoL with eosinophilia	0 (0.0%)
		Acute monocytic leukemia	2 (4.4%)
		Acute erythroid leukemia	0 (0.0%)
		Acute megakaryoblastic leukemia	0 (0.0%)
Mutations		*FLT3*-*ITD*/*NPM1*_*mut*_/*CEBPA*_*mut*_	9/3/1
Induction therapy outcome		Complete remission after 1st induction	24
		Complete remission after 2nd induction	6
		Complete remission after 3rd induction	3
		Mortality (1st/2nd/3rd induction/consolidation)	5/4/3/0
Post-consolidation treatment		Allogenic hematopoietic stem cell transplant	30
		Maintenance	3
Risk		Favorable risk	7 (15.6%)
		Intermediate risk I	10 (22.2%)
		Intermediate risk II	8 (17.8%)
		Unfavorable risk	20 (44.4%)

*NPM1*_*mut*_ mutated nucleophosmin, *CEBPA*_*mut*_ CCAAT/enhancer-binding protein alpha, *FLT3*-*ITD* internal tandem duplication of FMS-like tyrosine kinase 3

All patient samples were collected with the approval of the Ethics Committee at the Medical University of Bialystok and with a written informed consent form in accordance with the Declaration of Helsinki. AML patients were treated in the Hematology Department of the Medical University of Bialystok from 2008 to 2016 with 7-day induction chemotherapy regimens corresponding to the standard therapy based on the Polish Adult Leukemia Group: cytarabine was delivered as a continuous IV infusion for seven consecutive days at a dose of 200 mg/m^2^, while anthracycline for three consecutive days as an IV push at a dose of 50 mg/m^2^, and cladribine was administered for 5 days as an IV push at a dose of 5 mg/m^2^ (DAC schedule) [35]. Following evaluation of the induction response, non-responding patients were given re-induction protocol therapy of cytarabine, cladribine, filgrastim, and mitoxantrone (CLAG-M) and/or idarubicin, cytarabine, and etoposide (ICE) [36–38]. Consolidation therapies for patients who achieved complete remission consisted of cytarabine and mitoxantrone first, then a high dose of cytarabine, and finally either allogenic hematopoietic stem cell transplantation or maintenance therapy.

### Combined bisulfite restriction analysis

Genomic DNA (gDNA) from whole peripheral blood mononuclear cells (MNCs) of AML patients and from human cord blood MNCs (CBMNCs; Cleveland Cord Blood Center, Cleveland, OH) was purified using the DNA Mini Kit (Qiagen, Germantown, MD) and subjected to bisulfite treatment using the EpiTect Bisulfite Kit (Qiagen). Sequences of bisulfite-treated genomic DNA (BSgDNA) were amplified using specific oligo primers and the following thermocycler conditions: for AmpliTaq DNA Polymerase (Applied Biosystems, Waltham, MA)—(95 °C for 2 min, annealing temperature for 1 min, 72 °C for 1 min) ×  1 cycle, (95 °C for 30 s, annealing temperature for 1 min, 72 °C for 1 min) ×  cycle number, and (72 °C for 10 min) ×  1 cycle; for AmpliTaq Gold DNA Polymerase (Applied Biosystems)—(95 °C for 8 min) × 1 cycle, (95 °C for 2 min, annealing temperature for 1 min, 72 °C for 1 min) × 2 cycles, (95 °C for 30 s, annealing temperature for 1 min, 72 °C for 1 min) × cycle number, and (72 °C for 10 min) × 1 cycle. Primer sequences for each locus are listed in Table 2. The annealing temperatures and cycle numbers for each amplicon are listed in Table 3. Primer binding locations are listed in Table 4.

**Table 2:** Primer list for PCR of BSgDNA

Locus	1st PCR primer pair (5′-3′)	2nd PCR primer air (5′-3′)
*IGF2-H19* ICR	F–AGGTGTTTTAGTTTTATGGATGATGG [47] R–TCCCATAAATATCCTATTCCCAAATAACC [47]	F–TGTATAGTATATGGGTATTTTTGGAGGTTT [47] R–TCCCATAAATATCCTATTCCCAAATAACC [47]
*DLK1-MEG3* IG-DMR (CG6)	F–TGGGAATTGGGGTATTGTTTATAT R–AAACAATTTAACAACAACTTTCCTC	F–GTTAAGAGTTTGTGGATTTGTGAGAAATG [39] R–CTAAAAATCACCAAAACCCATAAAATCAC [39]
*MEG3* DMR (CG7)	F–TTATTTTTTTGAATAATAAGAGAAAGTATG R–CTCATTTCTCTAAAAATAATTAACC	F–TTATTTTTTTGAATAATAAGAGAAAGTATG R–CCCCAAATTCTATAACAAATTACT [39]
*MEG3* promotor (CG9)	F–TGAGGAAGTAGGGGTTTATAGAGAG R–AACCCTACAACCCCACAAAA [39]	F–GGAGAGTGGGGTTTATTGTGAA [39] R–AACCCTACAACCCCACAAAA [39]
*MEG3* intragenic (CG8)	F–GTTTGAGATTTGTTGGGTATTT [39] R–AATTTAACTAACAAATCACAAATATTAACT	F–GTTTGAGATTTGTTGGGTATTT [39] R–AATTTAACTAACAAATCACAAATATTAACT
*ZAC* DMR	F–GGGGTAGTYGTGTTTATAGTTTAGTA [48] R–CRAACACCCAAACACCTACCCTA [48]	F–GGGGTAGTYGTGTTTATAGTTTAGTA [48] R–CRAACACCCAAACACCTACCCTA [48]
*PEG1* DMR	F–TTGTTGGTTAGTTTTGTAYGGTT [47] R–AAAAATAACACCCCCTCCTCAAAT [47]	F–TTGTTGGTTAGTTTTGTAYGGTT [47] R–CCCAAAAACAACCCCAACTC [47]
*PEG3* DMR	F–AAAAGGTATTAATTATTTATAGTTTGGT [49] R–AAAAATATCCACCCTAAACTAATAA [49]	F–AAAAGGTATTAATTATTTATAGTTTGGT [49] R–AAAAATATCCACCCTAAACTAATAA [49]

**Table 3:** PCR conditions for amplifying BSgDNA

Locus	1st PCR condition			2nd PCR condition			COBRA
	Polymerase	Anealing temperature (°C)	Cycles	Polymerase	Anealing temperature (°C)	Cycles	Restriction enzyme
*IGF2-H19* DMR	AmpliTaq	55	35	AmpliTaq	55	35	BstUI
*DLK1-MEG3* IG-DMR (CG6)	AmpliTaq	55	35	AmpliTaq	55	35	BstUI
*MEG3* DMR (CG7)	GoldTaq	57	40	GoldTaq	57	40	TaqI
*MEG3* promotor (CG9)	AmpliTaq	57	35	AmpliTaq	57	35	TaqI
*MEG3* intragenic (CG8)	AmpliTaq	55	35	AmpliTaq	55	35	BstUI
*ZAC* DMR	GoldTaq	55	35	GoldTaq	55	38	BstUI
*PEG1* DMR	AmpliTaq	55	35	AmpliTaq	55	38	TaqI
*PEG3* DMR	AmpliTaq	55	35	AmpliTaq	55	35	BstUI

**Table 4:** Nucleotide coordinates for primers and *DLK1*-*MEG3* CTCF-binding sites

Locus	Chromosome	Nucleotide coordinates
*IGF2-H19* ICR	11	1999842–2000072
*DLK1-MEG3* IG-DMR (CG6)	14	100810848–100811276
*MEG3* DMR (CG7)	14	100825668–100825999
*MEG3* promotor (CG9)	14	100823942–100824184
*MEG3* intragenic (CG8)	14	100828129–100828322
*ZAC* DMR	6	144132370–144132521
*PEG1* DMR	7	130492229–130492444
*PEG3* DMR	19	56840361–56840682
*DLK1*-*MEG3* CTCF site B	14	100824015–100824074
*DLK1*-*MEG3* CTCF site D	14	100825725–100825784
*DLK1*-*MEG3* CTCF site F	14	100828145–100828204

Combined bisulfite restriction analysis (COBRA) of each amplicon was carried out on each amplicon via restriction enzyme digestion using either TaqI or BstUI (New England Biolabs, Ipswich, MA) using the following thermocycler conditions: 60 °C for 2 h for BstUI and 65 °C for 2 h for TaqI. The digested amplicons were separated by electrophoresis in agarose gels, and densitometric analysis of ethidium bromide-labeled bands was performed on photographed agarose gels using ImageJ software (National Institutes of Health). The specific COBRA conditions for each amplicon are listed in Table 3. Additional file 1**:** Figure S1 illustrates the quantitation method used for COBRA and includes all gels used for COBRA analyses. The median (range) values of methylation of studied CpG sites are presented in Table 5.

**Table 5:** The median (range) values of methylation of studied CpG sites and chosen gene expression

Analysis	AML patient MNCs Median (range) [number of patient]	Control MNCs Median (range) [number of sample]
Methylation (%)		
CG6	88.28 (5.00–99.53) [44]	60.89 (48.75–70.89) [9]
CG7	74.45 (32.65–97.69) [42]	60.21 (24.98–72.70) [9]
CG8	91.91 (30.43–99.65) [44]	65.65 (60.11–82.36) [9]
CG9	55.34 (7.86–91.73) [42]	46.53 (39.23–60.13) [8]
*ZAC*	49.24 (18.96–71.97) [42]	62.91 (46.50–78.97) [8]
*IGF2*-*H19*	70.20 (40.97–99.56) [45]	78.12 (68.64–86.20) [9]
*PEG3*	67.18 (15.95–98.10) [42]	67.41 (41.82–79.71) [9]
*PEG1*	56.29 (23.08–95.13) [45]	66.74 (57.85–68.91) [9]
Expression (∆Ct)		
*DLK1* (× 10^−7^)	50.98 (0.88–161,542.28) [29]	
*MEG3* (× 10^− 6^)	34.189 (1.36–19,339.85) [29]	
*DLK1*/*MEG3* (× 10^− 2^)	10.31 (0.20–1121.10) [29]	

### Real-time quantitative PCR

Total RNA was purified from whole peripheral blood MNCs of AML patients using TRIZOL (Life Technologies, Waltham, MA), and 2500 ng of RNA was transcribed to cDNA using Superscript VILO (Life Technologies). Gene expression analysis was carried out at least twice in duplicate using 2 ng of cDNA, 12.5 μL SYBR Select Master Mix (Applied Biosystems), and 150 nM forward and reverse primer sequences in 25 μL reaction mixtures. Real-time quantitative PCR (RT-qPCR) was performed using a 7500 Fast Real-Time PCR system (Applied Biosystems), and results were quantified using the ΔΔCt method. The following amplification conditions were carried out for real-time quantitative PCR (RT-qPCR) of each reaction mixture: 95 °C (15 s), 45 cycles at 95 °C (15 s), and 60 °C (1 min). *B2M* was used as a control gene. Primer sequences for each gene are listed in Table 6. The median (range) values of the chosen gene expression are presented in Table 5.

**Table 6:** Primer list for RT-qPCR

Gene	Forward primer (5′-3′)	Reverse primer (5′-3′)
*DLK1*	GCGAGGATGACAATGTTTGCA	GGTTCTCCACAGAGTCCGTGAA
*MEG3*	ATCCCGGACCCAAGTCTTCT	CCACATTCGAGGTCCCTTCC
*B2M*	TGACTTTGTCACAGCCCAAGATA	AATGCGGCATCTTCAAACCT

### Statistical analysis

GraphPad Prism 7 (GraphPad, La Jolla, CA), GraphPad InStat 3 (GraphPad), and SPSS software (IBM Corporation, Armonk, NY) were used for the statistical analysis. The Mantel-Cox test was used to analyze patient survival data with use of the Bonferroni post hoc analysis noted where appropriate (GraphPad Prism). Normal distribution of samples was tested using D’Agostino-Pearson normality test (GraphPad Prism). Individual gene expression, methylation, risk group, FMS-like tyrosine kinase 3 (*FLT3*) mutation, and patient first-round induction therapy response data were analyzed using the Mann-Whitney test or Kruskal-Wallis test and presented as mean ± SEM (GraphPad Prism). Analyses based on nucleophosmin 1 (*NPM1*) and CCAAT/enhancer-binding protein alpha (*CEBPA*) mutation status were omitted due to the limited number of patients. Pooled methylation data was analyzed with gene expression data using the Kruskal-Wallis test and presented as mean ± SEM (GraphPad Prism). Correlation data were obtained using Spearman’s rank-order correlation (GraphPad Prism) and the point-biserial correlation (SPSS Software). SPSS software was used for the Cox regression analysis (both univariable and multivariable analyses) and for the testing of a Cox proportional hazard assumption. Multicollinearity of samples was analyzed using GraphPad InStat software. All tests were performed as two-sided tests, and differences between sample sets were considered significant for *p* < 0.05.

## Results

### Increased methylation at the *MEG3* promotor region is found in AML patients with better overall survival

**Fig. 2:**
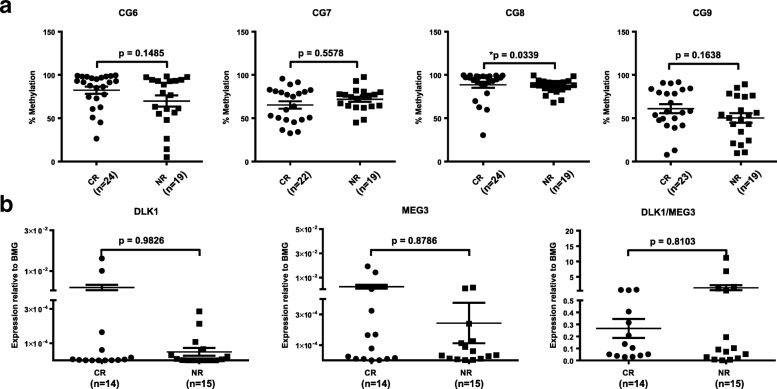
Differences in DLK1-MEG3 CpG site methylation and imprinted gene expression in AML MNCs based on patient response to first-round induction therapy. **a** Mann-Whitney analysis of AML patients’ response to treatment depending on the methylation values for particular CpG site amplicons. CR, complete remission; NR, non-responders. *p < 0.05. **b** Mann-Whitney analysis of AML patients’ response to treatment depending on DLK1, MEG3, and DLK1/MEG3 expression ratio values obtained by RT-qPCR. CR, complete remission; NR, non-responders

**Fig. 3:**
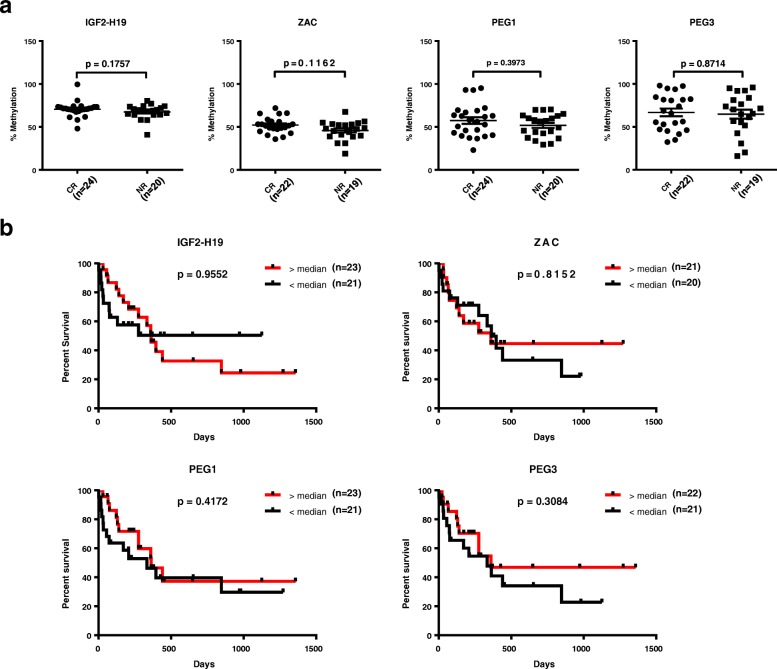
Patient response to first-round induction therapy and overall survival based on DMR methylation at imprinted gene network loci in AML MNCs. **a** Mann-Whitney analysis of AML patients’ response to treatment depending on the methylation values for particular imprinted gene loci DMR amplicons. CR, complete remission; NR, non-responders. **b** Analysis of AML patients’ survival depending on the median methylation values for particular imprinted gene loci DMR amplicons using the Mantel-Cox test

To investigate the relationship between methylation at the *DLK1*-*MEG3* locus and patient outcomes in AML, we assessed the methylation of four CpG sites within this locus (Fig. 1a) in AML patient peripheral blood mononuclear cells (MNCs) and compared our results with patient overall survival (OS) and first-round induction therapy response (IR). Previous methylation analyses of 9 CpG sites within *DLK1*-*MEG3* (CG1–9) in uniparental disomy patients revealed that CG6 and CG7, but not CG8 or CG9, are paternally imprinted [39]. In addition, at least seven CTCF-binding sites exist within *DLK1*-*MEG3* (sites A–G) [40], and three of these CTCF-binding sites are located within CG7 (site D), CG8 (site F), and CG9 (site B) (Table 4**)**. Together, these studies allowed us to analyze one confirmed non-imprinted CpG site which contains no known CTCF-binding site (CG6), one imprinted CpG site which contains a CTCF-binding site (CG7), and two non-imprinted CpG sites which each contain one CTCF-binding site (CG8, CG9). Our COBRA analyses of CG6–9 revealed that patients with increased methylation at CpG sites within the *MEG3* promotor region (CG7 and CG9) had significantly longer OS (Fig. 1a, b). We also utilized Spearman’s rank-order correlation to determine the correlation between methylation at CG6–9. The correlation coefficient (rho) indicates the strength and direction of the relationship between two variables, where − 1 and 1 indicate strong correlations (negative and positive, respectively) and 0 indicates a lack of correlation. Our analysis indicated that methylation at CG6 and CG8 was correlated (rho = 0.4121, *p* = 0.0060) but did not impact patient OS (Fig. 1b). In addition, slightly increased methylation at CG8 was observed in patients who achieved complete remission following first-round induction therapy (Fig. 2a). Interestingly, by pooling the methylation results at each CpG site within the *DLK1*-*MEG3* locus for each patient, we determined that patients with increased methylation at this locus had significantly longer OS than patients with lower methylation at this locus (Fig. 1c). Moreover, we found no significant contributions from the *IGF2*-*H19*, *ZAC*, *PEG1*, or *PEG3* locus on the IR (Fig. 3a) or OS (Fig. 3b) of AML patients. Taken together, these data highlight CpG sites within the *MEG3* promotor region as potential prognostic factors for AML patient OS and suggest that *DLK1*-*MEG3* imprinted gene dysregulation may explain this phenomenon.

### Increased expression of imprinted genes at the *DLK1*-*MEG3* locus is found in AML patients with worse overall survival

**Fig. 4:**
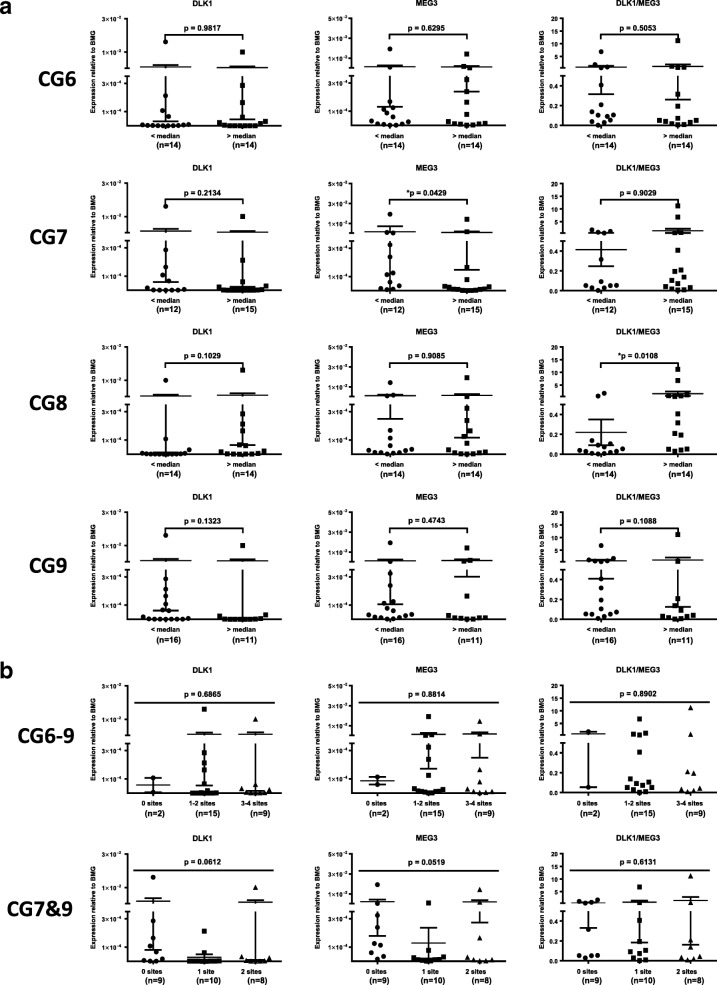
Relationship between *DLK1*-*MEG3* CpG site methylation and imprinted gene expression in AML MNCs. **a** Mann-Whitney analysis of *DLK1* and *MEG3* expression in AML MNCs based on the methylation values for particular CpG site amplicons. *p < 0.05. **b** Kruskal-Wallis test of *DLK1* and *MEG3* expression in AML MNCs based on the pooled CG6–CG9 or CG7 and CG9 amplicon median methylation results. Groups represent the number of CpG site amplicons out of 4 (CG6–9) or 2 (CG7 and CG9) analyzed which exhibited increased methylation relative to their respective median values

To gain insight into the relationship between *DLK1*-*MEG3* imprinted gene expression and AML patient outcomes, we probed *DLK1* and *MEG3* expression in AML MNCs and found no significant differences in the expression of these genes based on IR (Fig. 2b). As mentioned before, *DLK1* overexpression enhances the proliferation of leukemic cells [41], and we found increased *DLK1* expression in patients with significantly shorter OS (Fig. 1d). Interestingly, no significant differences in *DLK1* expression were found based on individual (Fig. 4a) or pooled (Fig. 4b) CpG site methylation, consistent with the known mechanism of *DLK1* regulation in AML [26]. On the other hand, we observed a trend for shorter OS in patients with increased expression of the tumor suppressor [27] *MEG3* (Fig. 1d). Our pooled results for *DLK1* and *MEG3* expression also revealed that patients with increased expression of *DLK1*, *MEG3*, or both genes had significantly shorter OS than patients with lower expression of these genes (Fig. 1d). We found significant differences in *MEG3* expression based on CG7 methylation (Fig. 4a) and a significant correlation between them (rho = − 0.3846, *p* = 0.0476), despite our survival results for CG7 methylation (Fig. 1b). *DLK1* expression also correlated with *MEG3* expression (rho = 0.7163, *p* < 0.0001) and the *DLK1*/*MEG3* expression ratio (rho = 0.6227, *p* = 0.0003). In addition, significant differences in the *DLK1*/*MEG3* expression ratio were found based on CG8 methylation (Fig. 4a) along with a significant correlation between them (rho = 0.4532, *p* = 0.0154). No significant differences in the *MEG3* expression or *DLK1*/*MEG3* expression ratio were found based on pooled methylation results (Fig. 4b). Collectively, these data indicate that *DLK1* is a potential prognostic factor for AML patient OS and the tumor-suppressing abilities of *MEG3* may be overwhelmed by *DLK1* expression and/or CpG site-specific signatures of its downstream miRNAs [33].

### Multivariable analysis confirms CG9 methylation as an independent prognostic factor for survival

**Fig. 5:**
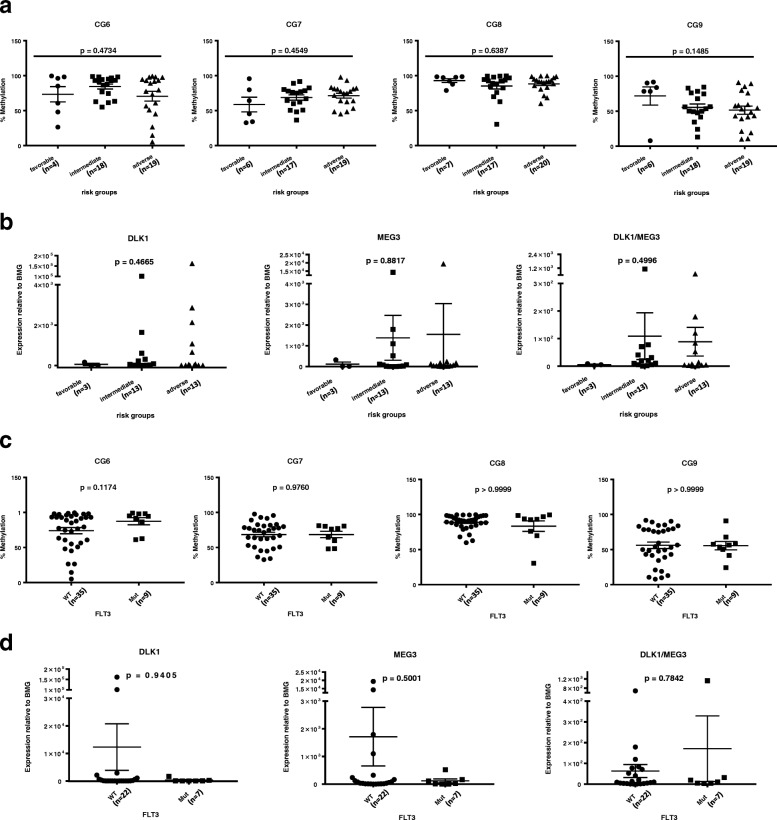
Differences in *DLK1*-*MEG3* CpG site methylation and imprinted gene expression based in AML MNCs based on patient risk group and *FLT3* mutation status. Kruskal-Wallis (**a**) or Mann-Whitney (**c**) analysis of methylation values for particular CpG site amplicons depending on AML patients’ risk groups (**a**) or presence of *FLT3* mutation (**c**). WT, wild-type; Mut, mutated. Kruskal-Wallis (**b**) or Mann-Whitney (**d**) analysis of *DLK1* and *MEG3* and the *DLK1*/*MEG3* expression ratio in MNCs from AML patients based on their risk group (**b**) or presence of *FLT3* mutation (**d**). WT, wild-type; Mut, mutated

To examine the prognostic values of our methylation and expression results in AML survival, we compared them with already known survival predictors including cytogenetics (risk groups and *FLT3* mutation status), white blood count (WBC), lactate dehydrogenase (LDH) level, and age. Analyses based on nucleophosmin (*NPM1*) and CCAAT/enhancer-binding protein alpha (*CEBPA*) mutation status were omitted due to the limited number of patients. Significant correlations were observed between CG7 methylation and age (rho = − 0.3088, *p* = 0.0466) and between patient WBC and LDH level (rho = 0.4497; *p* = 0.0022). No significant differences in CpG site methylation or imprinted gene expression were found based on the patient risk group (Fig. 5a, b) or *FLT3* mutation status (Fig. 5c, d). Univariable analysis performed for all patients confirmed that risk group, IR, and LDH level are strong predictors of OS and identified CG7 methylation, CG9 methylation, and *DLK1* expression as new possible predictors of OS. Multivariable Cox proportional hazard models carried out independently for significant methylation (CG7 and CG9) and expression (*DLK1*) factors indicated that only CG9 methylation can be considered a prognostic factor for survival independent of risk group, IR, and LDH level (Table 7). In total, these data identify CG9 methylation specifically as an independent prognostic factor for AML patient OS and suggest that miRNAs mediate this phenomenon.

**Table 7:** Statistical analyses of overall survival

	Overall survival		
	HR	95% confidence interval	*p* value
Univariate analysis			
Demographic			
Sex (male vs female)	0.887	0.396–1.987	0.770
Age (< 60 vs 60+)	1.461	0.625–3.414	0.381
Cytogenetic/clinical factors			
Risk groups (favorable vs intermediate vs adverse)	2.325	1.183–4.569	0.014
FLT3 (WT vs mutated)	1.282	0.474–3.466	0.624
Response (NR vs CR)	0.213	0.089–0.509	0.001
WBC count (low vs high)	1.168	0.514–2.650	0.711
LDH level (low vs high)	0.242	0.101–0.584	0.002
Methylation			
CG6 (low vs high)	0.894	0.386–2.068	0.793
CG7 (low vs high)	0.380	0.157–0.917	0.031
CG8 (low vs high)	0.762	0.335–1.731	0.516
CG9 (low vs high)	0.331	0.136–0.805	0.015
Expression			
DLK1 (low vs high)	3.212	1.096–9.413	0.033
MEG3 (low vs high)	2.294	0.819–6.421	0.114
DLK1/MEG3 (low vs high)	0.668	0.247–1.808	0.428
Multivariate analysis			
Methylation			
CG7 (low vs high)	0.533	0.185–1.538	0.245
CG9 (low vs high)	0.294	0.092–0.935	0.038
Expression			
DLK1 (low vs high)	1.758	0.467–6.615	0.404

Low or high refers to values either lower or higher than median calculated for all AML patients included in this study. *HR* hazard rate, *FLT3* FMS-like tyrosine kinase 3, *NR* non-responders, *CR* complete remission

## Discussion

Multiple CpG sites within *DLK1*-*MEG3* house CCCTC-binding factor (CTCF)-binding DNA sequences [26, 40] which govern the expression of coding [26] and non-coding RNAs (ncRNAs) [33] from this locus. Our investigation into the relationship between select CpG site methylation and AML patient outcomes identified increased methylation at CG7 and CG9 in patients with longer OS. Interestingly, CG7 and CG9 both contain CTCF-binding DNA sequences [40] and are located within the *MEG3* promotor region, where increased methylation was previously found to predict poor OS for AML patients [42]. This disparity is likely explained by differences in methodology and CpG site selection given that, similar to our results, increased methylation at a region containing CG7 was also observed in longer-living patients with MDS or AML with myelodysplasia-related changes (AML-MR) [43]. Furthermore, our data for DMRs within other IGN loci uncovered no differences in patient outcomes based on their methylation, highlighting the significance of our observation for CG7 and CG9 in survival prediction. Thus, the imprinted CG7 and non-imprinted CG9 [44] are potential prognostic factors for AML patient OS, and future investigations may reveal CpG site methylation-dependent [33] miRNA signatures from this locus which promote cell stemness [16, 30, 31] and self-renewal [30].

Pioneering studies in bimaternal mouse models uncovered a requirement for the balanced expression of growth-promoting *DLK1* and growth-suppressing *MEG3* in embryonal growth [10] and a more specific requirement for Dlk1 in fetal hematopoiesis [14]. Accumulating evidence suggests the opposing roles of these genes on growth and development are mirrored in leukemias, where *DLK1* maintains cell stemness [25] and enhances the proliferation of leukemic cells [41] and *MEG3* suppresses leukemogenesis [27] and leukemic cell proliferation [28]. By analyzing the expression of *DLK1* and *MEG3* in AML patient MNCs, we determined that increased expression of these genes is found in patients with worse overall survival. While this is consistent with the growth-promoting effects of *DLK1* on leukemic cells, our data for *MEG3* alludes to the presence of underlying miRNA signatures from this locus which may overcome the tumor-suppressing abilities of *MEG3*. In support of this, the negative correlation we observed between CG7 methylation and *MEG3* expression is consistent with the recent work of Yao et al. [29] in AML patients from a Hainan population and supported by work by Merkerova et al. which observed increased *MEG3* expression in MDS/AML-MR patients with shorter OS [43]. Taken together, our data emphasizes the overlapping roles of paternally [25] and maternally expressed [16, 30] genes from the *DLK1*-*MEG3* locus in stemness maintenance and proliferation as potential drivers of leukemia progression.

Several lines of evidence point to the dysregulation of the *DLK1*-*MEG3* locus as a key component of leukemia development and progression. *DLK1* expression is elevated in the CD34^+^ cells and MNCs of myelodysplastic syndrome (MDS) patients and MNCs of AML patients, and increased levels of DLK1 are found in MDS patient sera [41]. *MEG3* expression is similarly decreased in AML patients [29], and methylation at CpG sites within the *DLK1*-*MEG3* locus is likely responsible for the abnormal expression of these genes [26, 29]. We therefore sought to delineate the unique contribution(s) of the *DLK1*-*MEG3* locus to AML patient OS, and univariable analyses validated our observations that CG7 methylation, CG9 methylation, and *DLK1* expression are potential prognostic factors for survival of this disease. The multivariable analysis further clarified these results to reveal CG9 methylation as an independent prognostic factor for AML patient OS. We found this particularly interesting because, unlike CG6 or CG7, CG9 is not imprinted [44]. We also found no correlations between CG9 and patient biomarkers or gene expression. Thus, we hypothesize that the prognostic value of CG9 methylation can be explained by its regulation of the *DLK1*-*MEG3* miRNA mega-cluster [33] via the chromatin-modifying CTCF protein [40]. In addition, emerging evidence points to small nucleolar RNAs (snoRNAs) from the *DLK1*-*MEG3* locus as potential mediators of leukemic cell proliferation [45] and differentiation [46]. As a pilot study, we recognize the limitations of a 45-patient cohort. However, the results of our CpG site selection strategy add to the mounting evidence that methylation at CTCF-binding sites is responsible for chromatin dysregulation of the entire *DLK1*-*MEG3* locus, and our data suggest that increased methylation at CG9 specifically exerts a protective role in AML. Future studies with large patient cohorts are necessary to delineate the CTCF-mediated chromatin organization at *DLK1*-*MEG3* and its role in AML development and prognosis.

## Conclusions

Taken together, our screenings of imprinted loci methylation and gene expression in AML patient MNCs highlight a unique role for the *DLK1*-*MEG3* locus in AML patient prognosis. To our knowledge, we are the first to compare methylation at confirmed *DLK1*-*MEG3* CpG sites with survival in a relatively large cohort of AML patients comprised of multiple AML subtypes. We identify methylation at the non-imprinted CpG site CG9 as a novel independent prognostic factor for survival in AML patients which implicates CTCF-mediated ncRNA regulation as a key determinant of AML patient survival. In particular, considering that the *DLK1*-*MEG3* locus is the home to over 40 miRNAs, many of which exhibit functional redundancies [16, 30], the assessment of unique miRNA signatures from this locus for their prognostic values represents a valuable pursuit. Future studies involving large patient cohorts are required to confirm our findings for CG9 and further clarify the prognostic impact of the role of chromatin organization at *DLK1*-*MEG3* in AML.

## Additional file

Additional file 1: Scheme of densitometry analysis. a Equal amounts of PCR product (treated and untreated with restriction enzyme, RE) were separated on a gel, and a picture of the gel was captured. Step (1) Selection of bands for analysis. Step (2) Intensity of the bands is translated to the surface of the corresponding peaks using ImageJ software. Step (3) Analysis of the surface of the peaks corresponding to particular bands and re-calculation of arbitrary units to percentages. In analysis 1, the surface of the “unmethylated” band from the re-treated sample is compared with the total amount of PCR product that was used for digestion. In analysis 2, the surface of the “unmethylated” band is compared to the total surface of all peaks in the digested sample. The mean of the methylation from both analyses is then used for further studies. Gels are shown for the COBRA analysis of CG6 (b), CG7 (c), CG8 (d), CG9 (e), ZAC (f), IGF2-H19 (g), PEG1 (h), and PEG3 (i). (PDF 1340 kb)
