# Supplementary material for: Increased methylation upstream of the MEG3 promotor is observed in acute myeloid leukemia patients with better overall survival
Source: Clin Epigenetics. 2019 Mar 15;11:50. doi: 10.1186/s13148-019-0643-z (PMC6419839; doi:10.1186/s13148-019-0643-z)

a **Supplementary Figure 1**

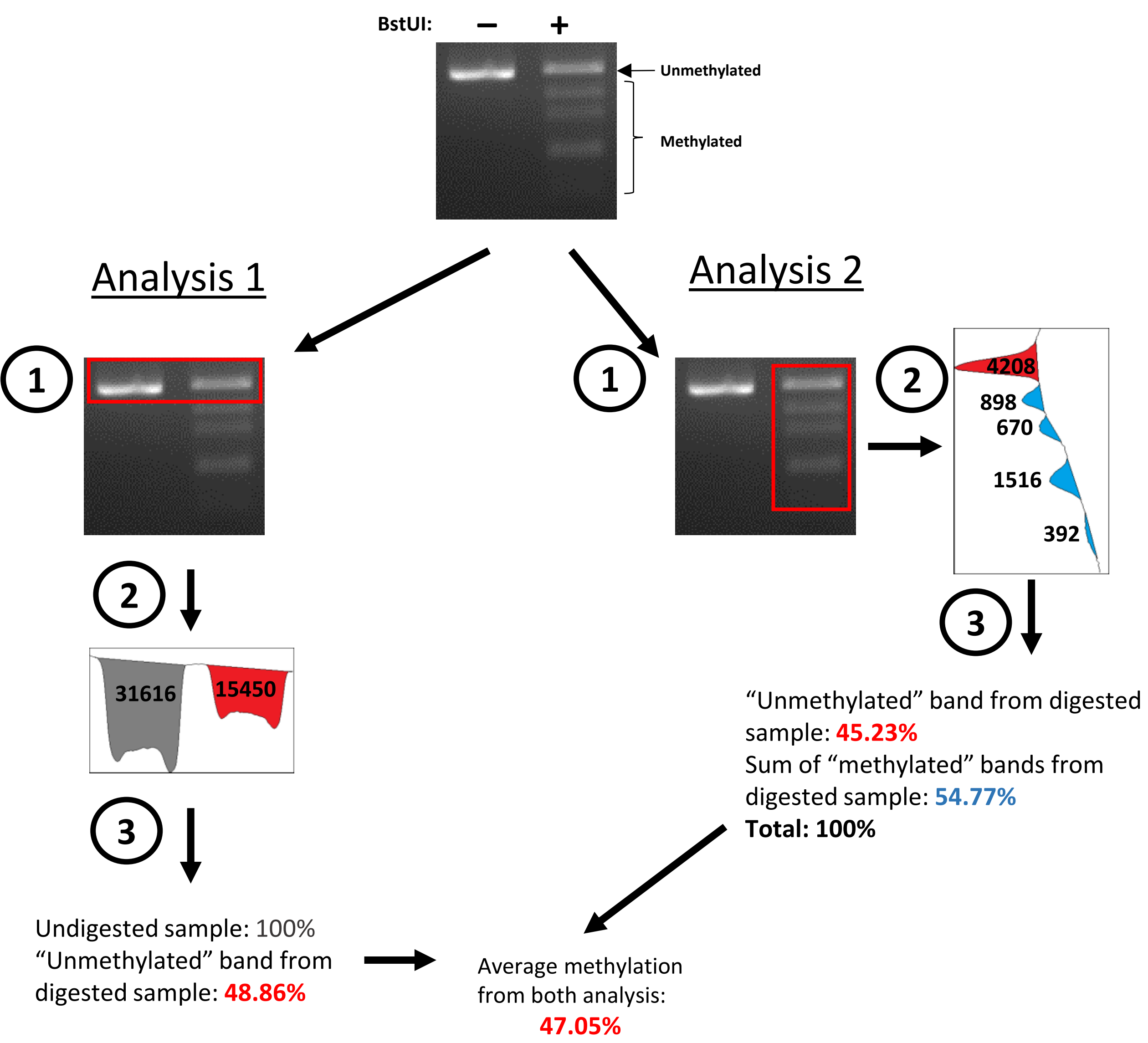

Supplementary Figure 1

Scheme of densitometry analysis. a Equal amounts of PCR product (treated and untreated with restriction enzyme, RE) were separated on a gel and a picture of the gel was captured. **Step (1)** Selection of bands for analysis. **Step (2)** Intensity of the bands is translated to the surface of the corresponding peaks using ImageJ software. **Step (3)** Analysis of the surface of the peaks corresponding to particular bands and recalculation of arbitrary units to percentages. In **Analysis 1**, the surface of the "unmethylated" band from the RE-treated sample is compared with the total amount of PCR product that was used for digestion. In **Analysis 2**, the surface of the "unmethylated" band is compared to the total surface of all peaks in the digested sample. The mean of the methylation from both analyses is then used for further studies. Gels are shown for the COBRA analysis of CG6 (**b**), CG7 (**c**), CG8 (**d**), CG9 (**e**), ZAC (**f**), IGF2-H19 (**g**), PEG1 (**h**), and PEG3 (**i**).

Supplementary Figure 1

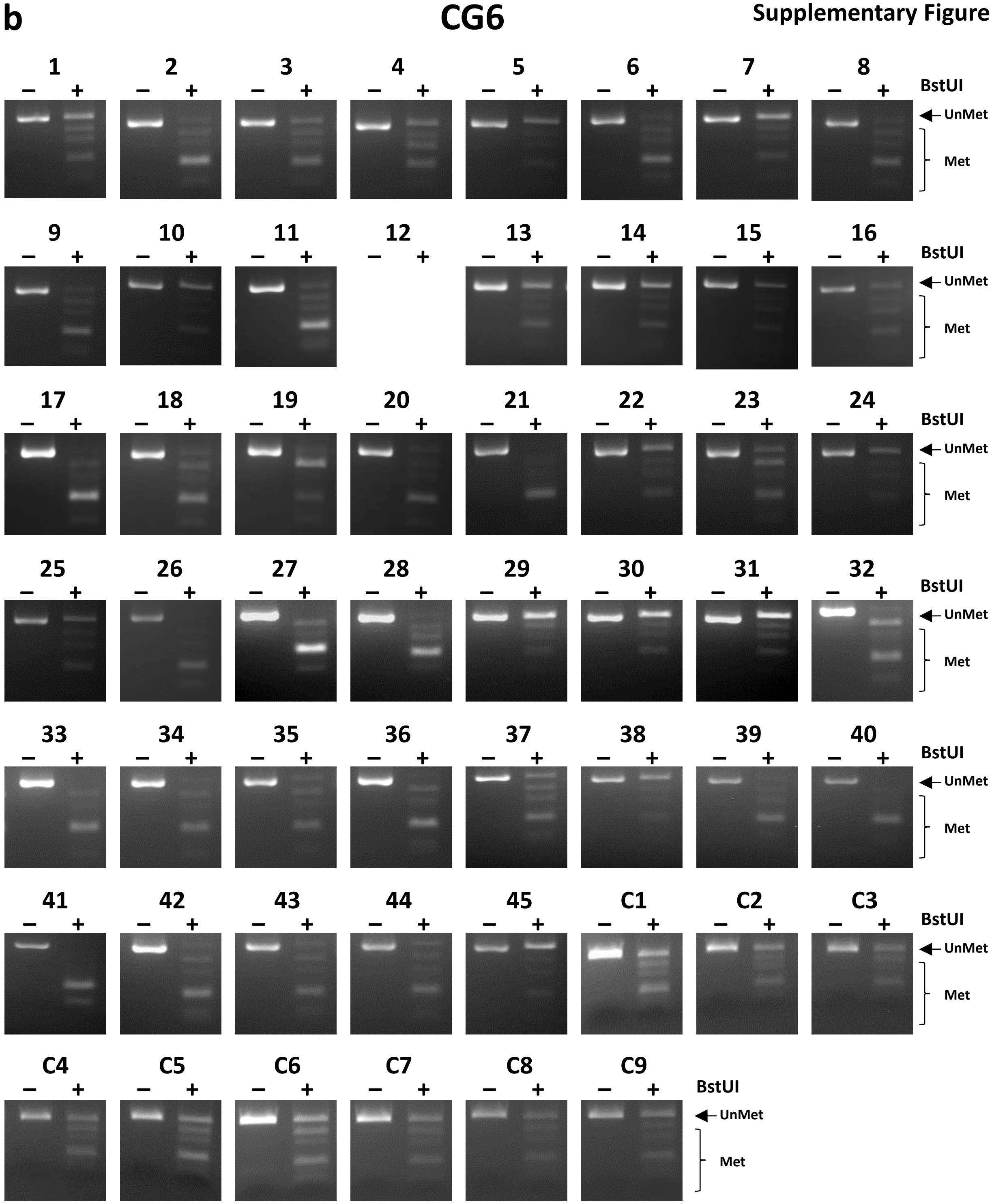

Supplementary Figure 1

c

CG7

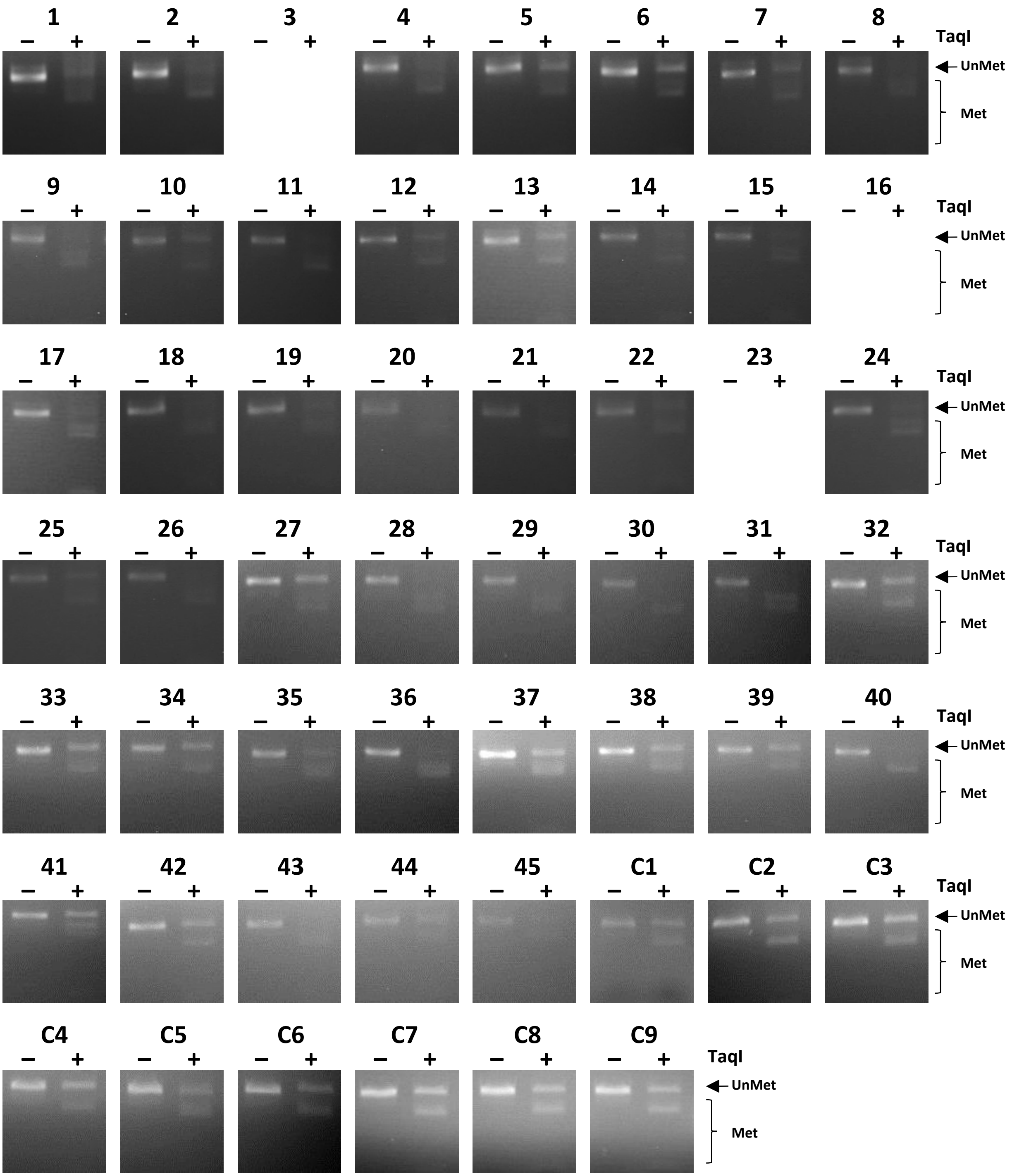

Supplementary Figure 1

d **CG8**

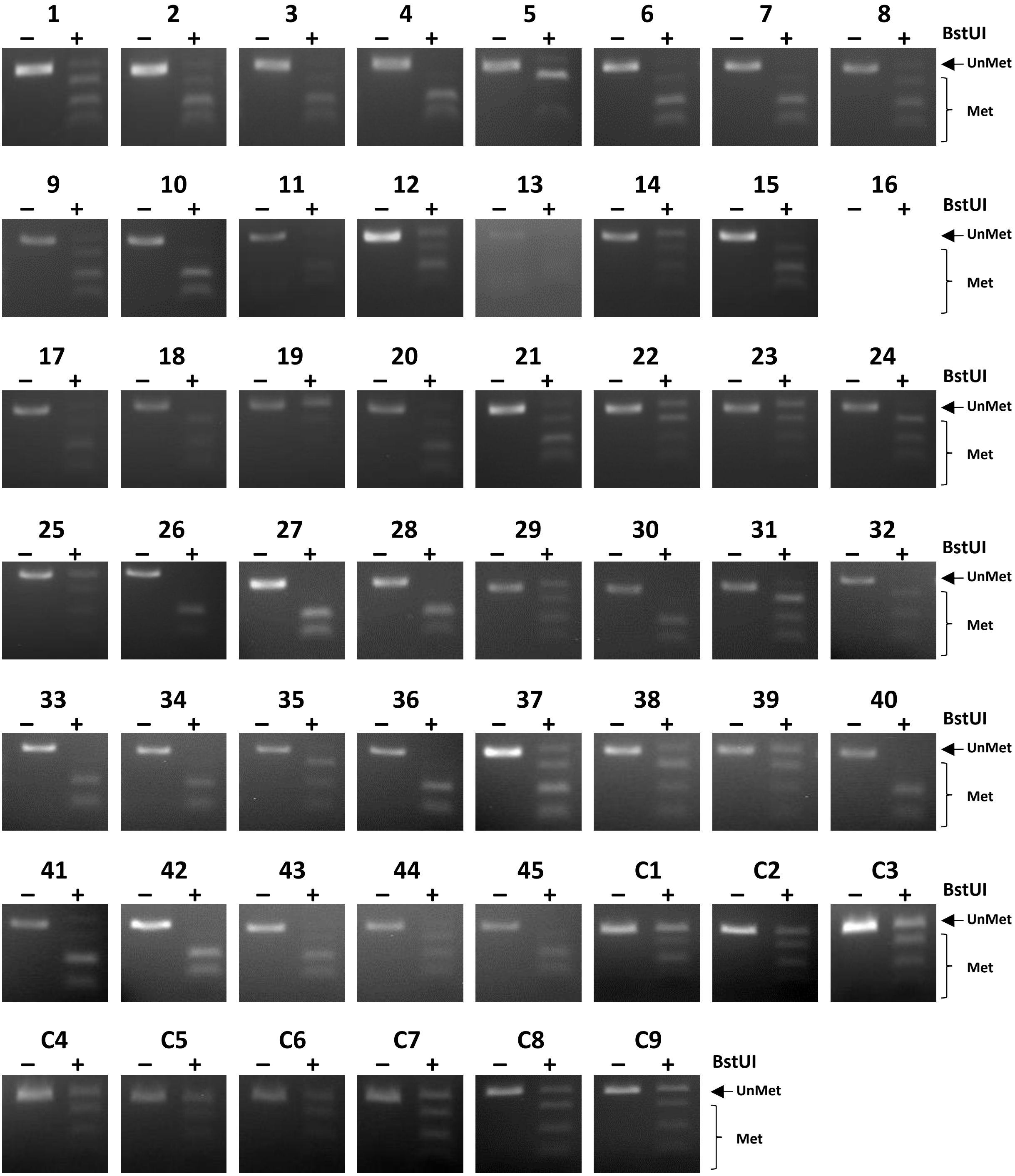

e

CG9

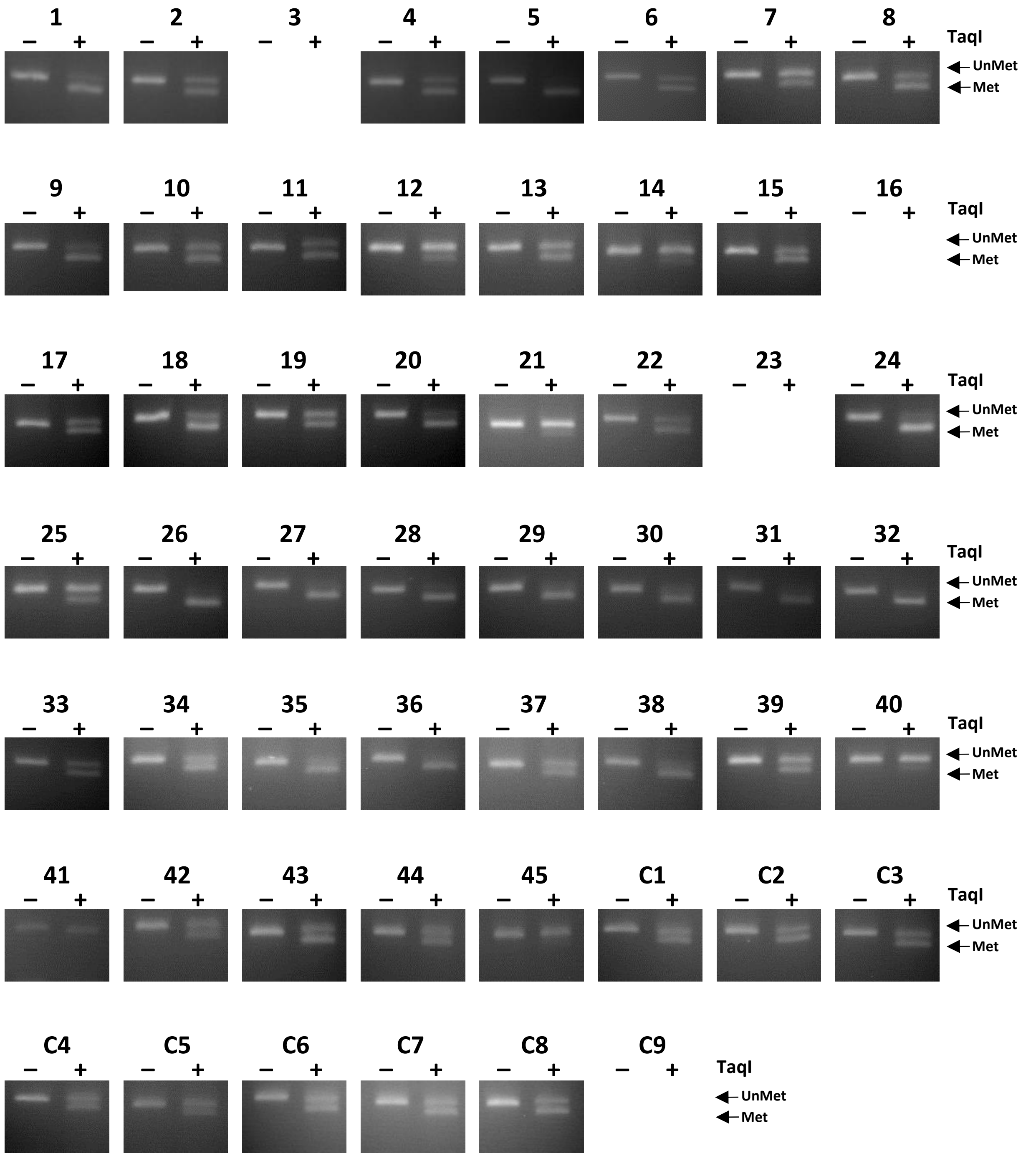
1 2 3 4 5 6 7 8
9 10 11 12 13 14 15 16
17 18 19 20 21 22 23 24
25 26 27 28 29 30 31 32
33 34 35 36 37 38 39 40
41 42 43 44 45 C1 C2 C3
C4 C5 C6 C7 C8 C9
− +
TaqI
UnMet
Met

Supplementary Figure 1

f ZAC

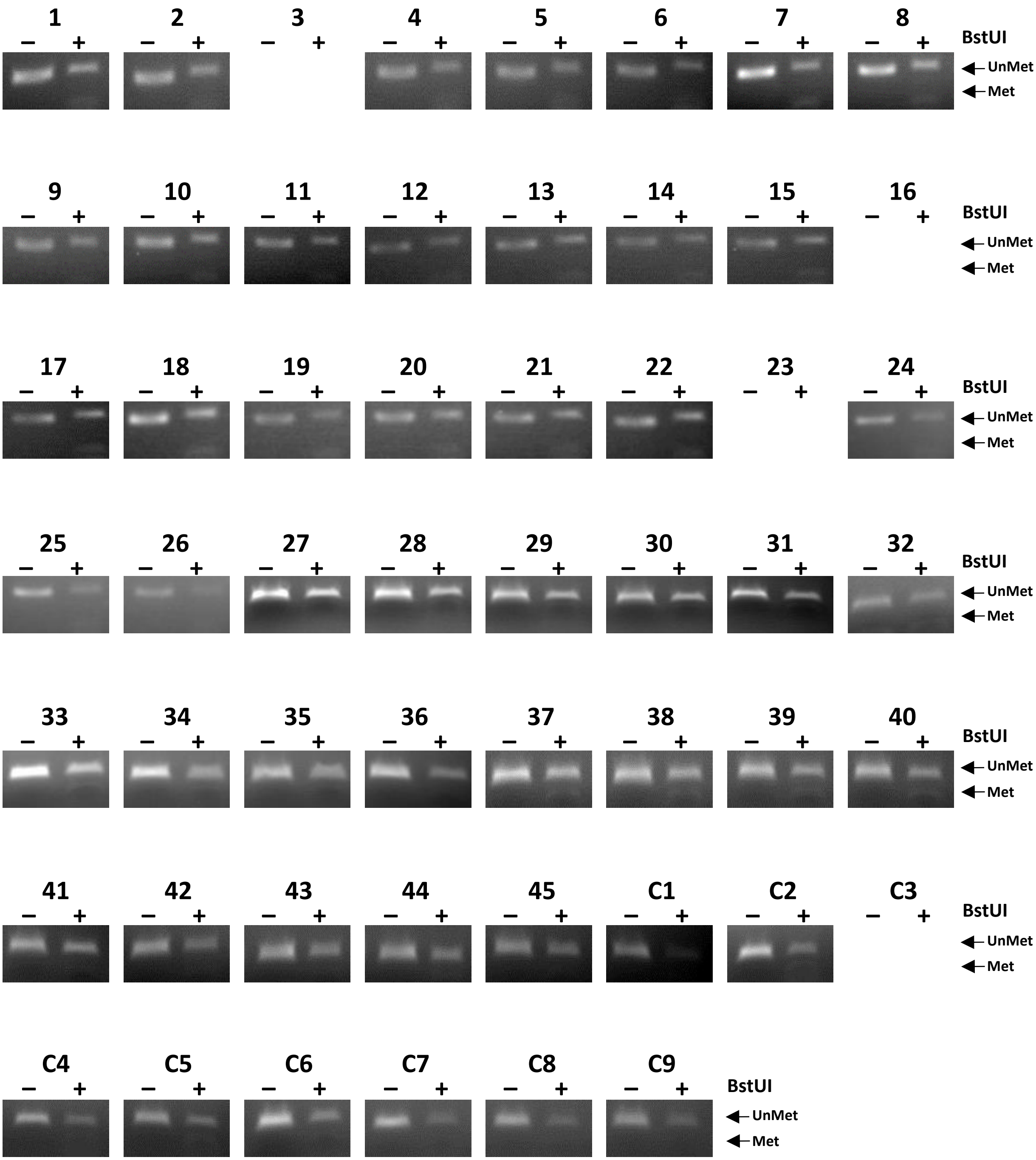

g

IGF2-H19

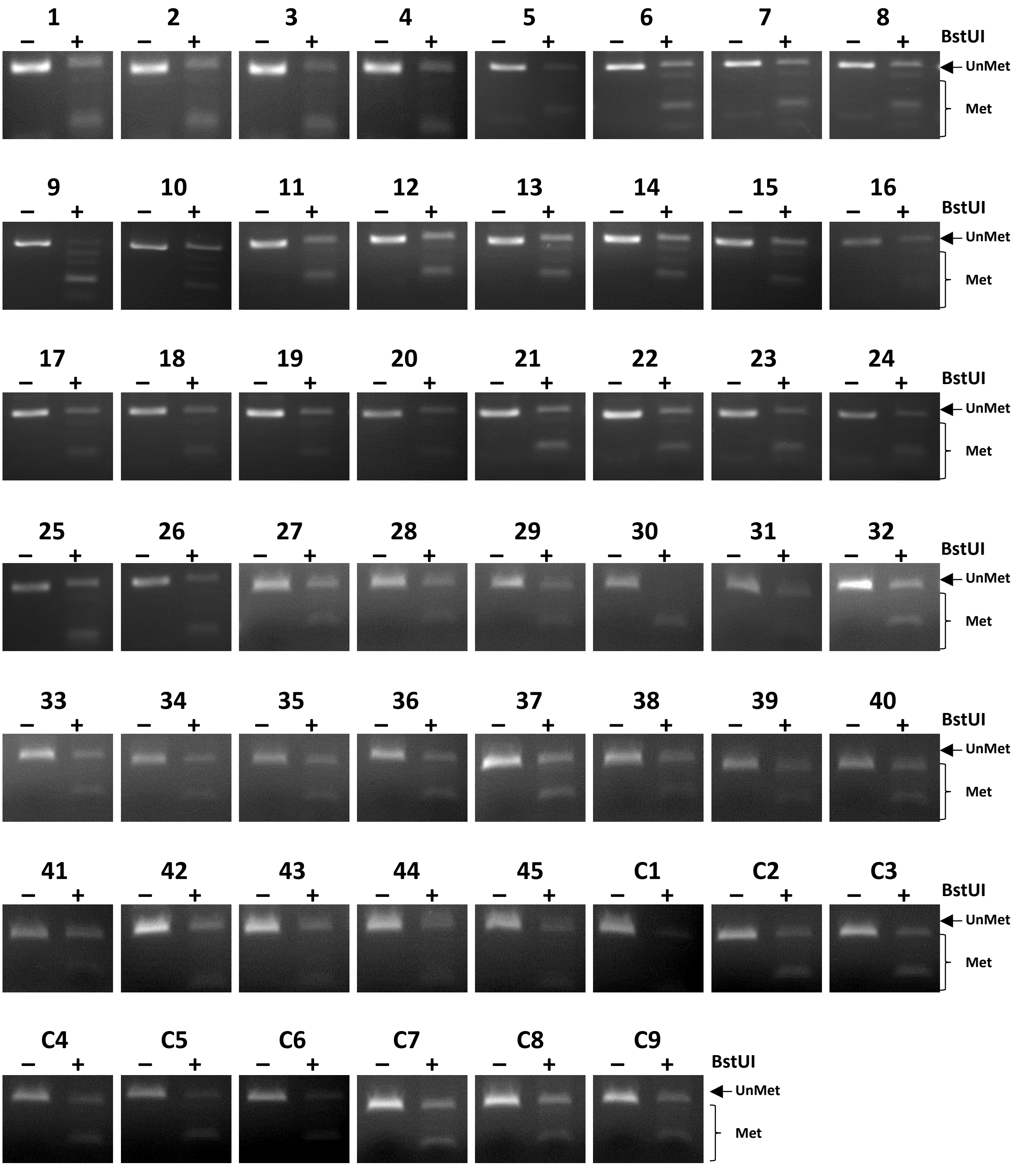

1
2
3
4
5
6
7
8
9
10
11
12
13
14
15
16
17
18
19
20
21
22
23
24
25
26
27
28
29
30
31
32
33
34
35
36
37
38
39
40
41
42
43
44
45
C1
C2
C3
C4
C5
C6
C7
C8
C9
− +
BstUI
UnMet
Met

h

PEG1

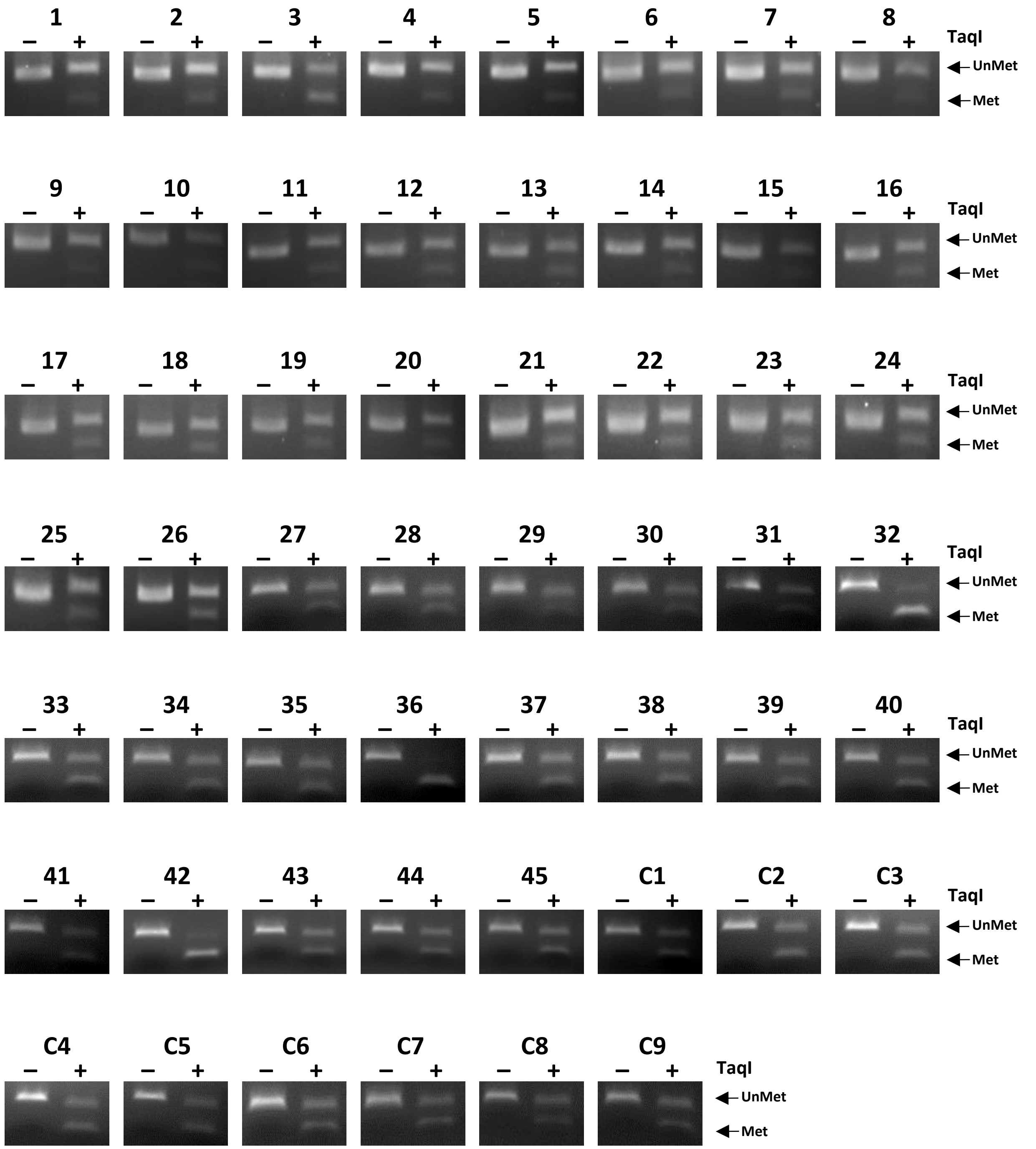
1 2 3 4 5 6 7 8
9 10 11 12 13 14 15 16
17 18 19 20 21 22 23 24
25 26 27 28 29 30 31 32
33 34 35 36 37 38 39 40
41 42 43 44 45 C1 C2 C3
C4 C5 C6 C7 C8 C9
− +
TaqI
UnMet
Met

i

PEG3

Supplementary Figure 1

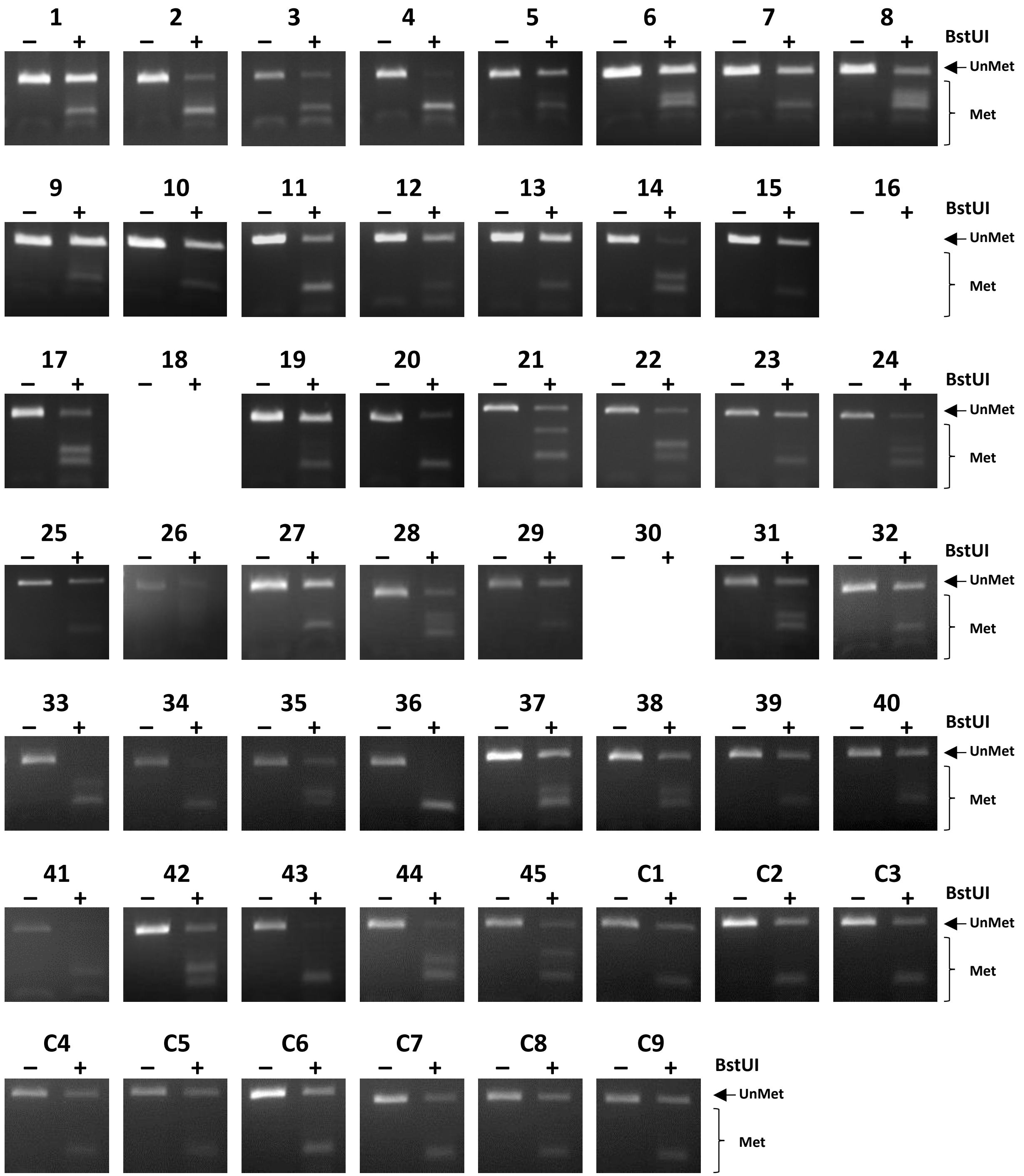

Supplement: Supplementary file 1 — Scheme of densitometry analysis. a Equal amounts of PCR product (treated and untreated with restriction enzyme, RE) were separated on a gel, and a picture of the gel was captured. Step (1) Selection of bands for analysis. Step (2) Intensity of the bands is translated to the surface of the corresponding peaks using ImageJ software. Step (3) Analysis of the surface of the peaks corresponding to particular bands and re-calculation of arbitrary units to percentages. In analysis 1, the surface of the “unmethylated” band from the re-treated sample is compared with the total amount of PCR product that was used for digestion. In analysis 2, the surface of the “unmethylated” band is compared to the total surface of all peaks in the digested sample. The mean of the methylation from both analyses is then used for further studies. Gels are shown for the COBRA analysis of CG6 (b), CG7 (c), CG8 (d), CG9 (e), ZAC (f), IGF2-H19 (g), PEG1 (h), and PEG3 (i). (PDF 1340 kb) [file 13148_2019_643_MOESM1_ESM.pdf]
